# Rare case of inflammatory abdominal aortic aneurysm with an aortocaval fistula

**DOI:** 10.1016/j.radcr.2023.09.075

**Published:** 2023-10-28

**Authors:** Richard Pham, Edwin Chou, Derek Breiner, Justin Wei, Veena Mandava, Duy Bui

**Affiliations:** University of California Riverside School of Medicine, Riverside, CA, USA

**Keywords:** Inflammatory abdominal aortic aneurysms, Aortocaval fistula, Aneurysm

## Abstract

Inflammatory abdominal aortic aneurysms (IAAA) are a distinct subcategory of abdominal aortic aneurysms that make up roughly 5%-10% of all abdominal aortic aneurysm (AAA) cases. Inflammatory AAA (IAAA) is distinguished from traditional atherosclerotic AAA by the triad of thickened aneurysm wall, extensive perianeurysmal/retroperitoneal fibrosis, and dense adhesions of adjacent abdominal organs. The purpose of this report is to examine the clinical course of a rare case of inflammatory abdominal aortic aneurysm with aortocaval fistula.

## Introduction

The perioperative morbidity and mortality rate is approximately 3-fold higher in inflammatory AAA in comparison to AAA. Therefore, distinguishing IAAA from traditional AAA is essential when considering treatment modalities and anticipated clinical outcomes. IAAA can differ from AAA in demographic, symptoms, pathology, and imaging characteristics. By understanding these differences, providers can appropriately diagnose and treat IAAA.

## Case presentation

A 63-year-old male presented initially with back pain and lack of bowel movements for the last 5 days. The patient has a 25 pack year smoking history. The patient denied any further medical or surgical history. The patient is unaware of any previous family medical history. Initial lab values show an elevated creatinine. Due to the patients presenting symptoms and extensive smoking history, AAA was initially suspected.

**Fig. 1:**
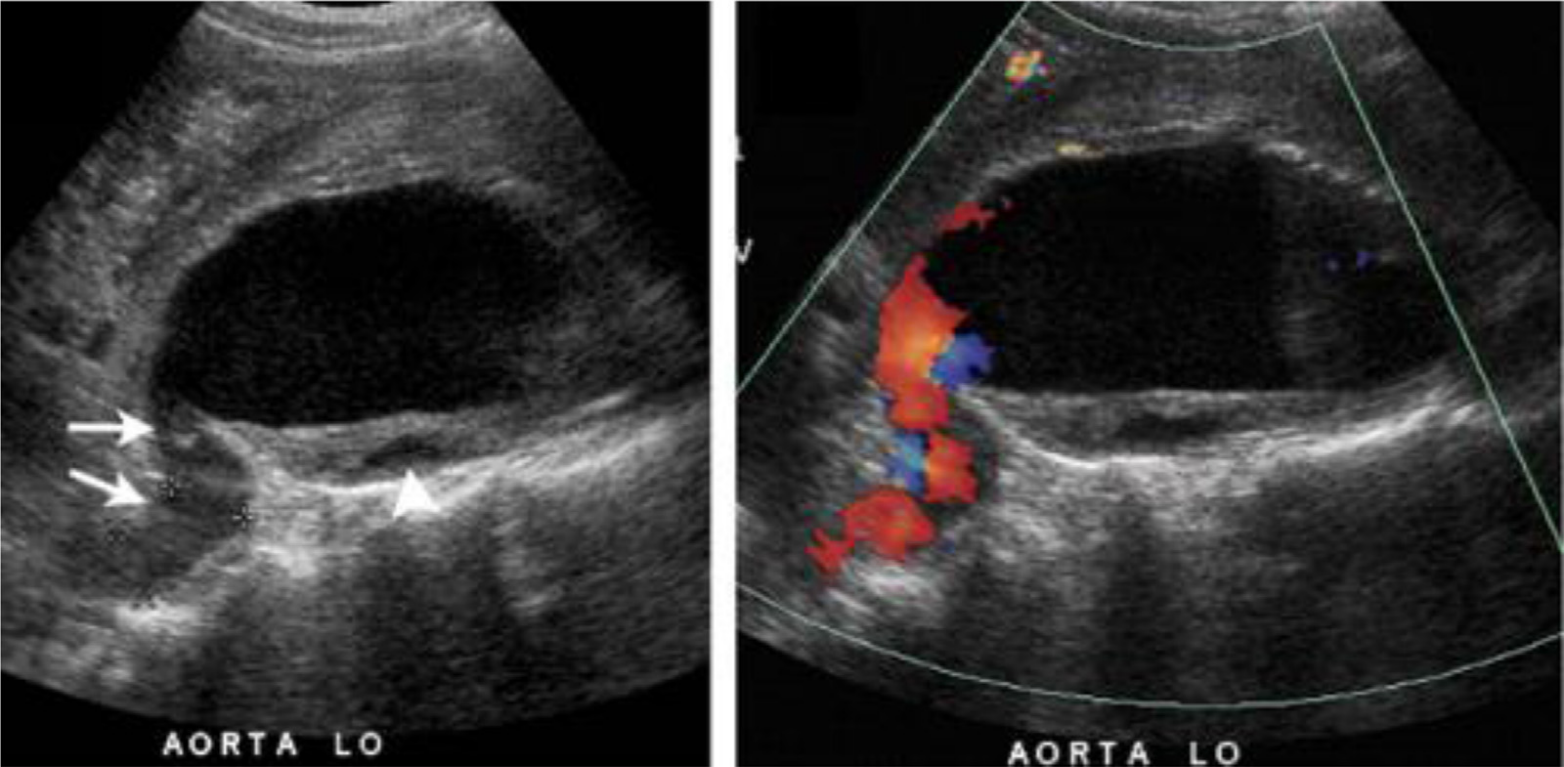
Bedside ultrasound revealed 8 cm AAA.

**Fig. 2:**
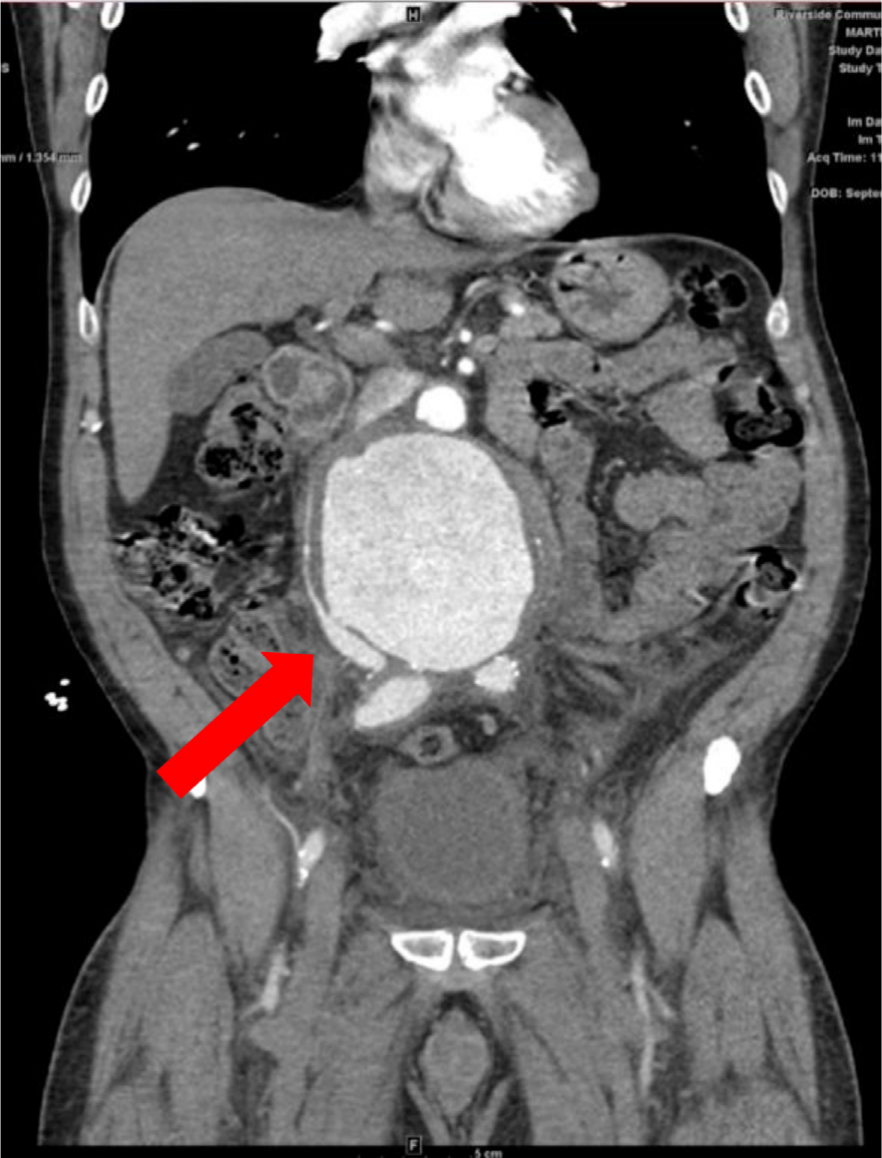
CTA demonstrates a contained rupture of the thickened and fibrotic aneurysmal wall into the adjacent vena cava.

**Fig. 3:**
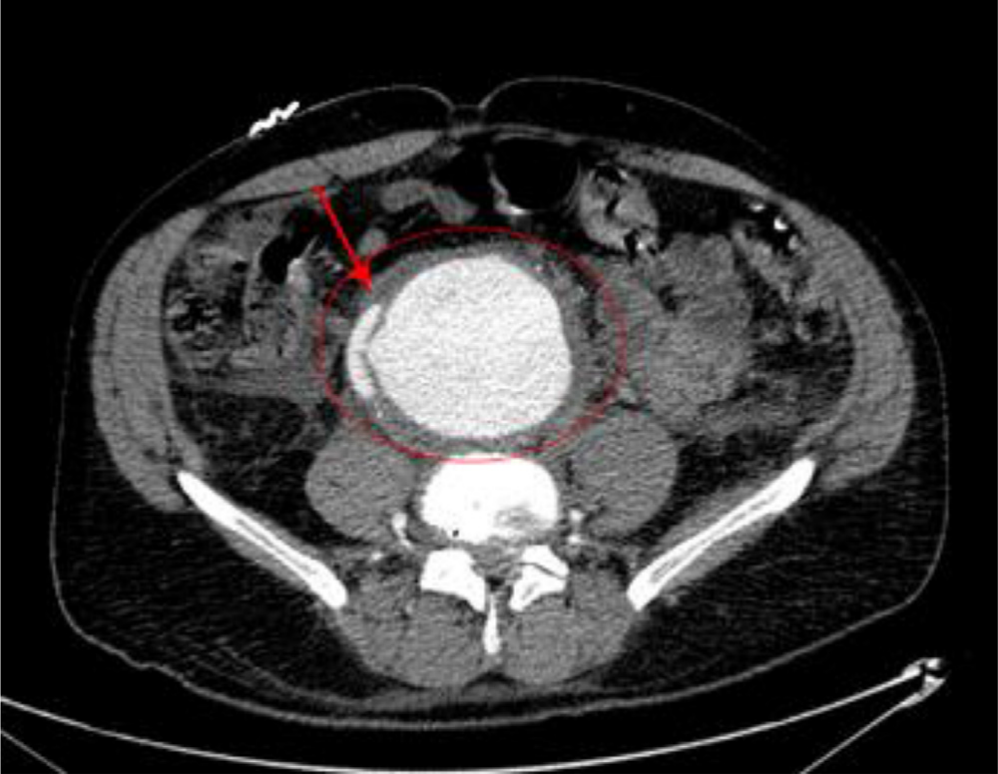
Axial CTA demonstrates fistulous tract between aorta and IVC.

Bedside ultrasound revealed an 8 cm AAA (Fig. 1). However, follow-up CTA demonstrates a contained rupture of the thickened and fibrotic aneurysmal wall into the adjacent vena cava (Fig. 2). Axial CTA demonstrated fistulous tract between aorta and IVC (Fig. 3). These imaging findings raised suspicion for inflammatory AAA, rather than traditional AAA, with a contained rupture into the adjacent inferior vena cava, causing an aortocaval fistula. The patient was immediately started on an esmolol drip. Due to the patient's symptoms and size of his aneurysm, the patient was sent to the OR for urgent open IAAA repair.

The patient was found to have an 11cm inflammatory AAA intraoperatively. The presence of extensive dense retroperitoneal adhesions and aortocaval fistula were also confirmed intraoperatively during the open AAA repair with Dacron graft. There were no complications during the patient's procedure. The patient was extubated the same day with vitals within normal limits. Despite initially concerning presentation and imaging findings, this 63-year-old male smoker did well postoperatively and was safely discharged on postoperative day 4. The patient was educated on the importance of smoking cessation and was scheduled for outpatient follow-up.

## Discussion

IAAA affects males at a significantly higher rate than females. In comparison to traditional atherosclerotic AAA, IAAA patients are typically 5-10 years younger. An estimated 77% of patients affected by IAAA are either previous or current smokers, making tobacco use one of the highest risk factors [1]. Genetic predisposition also plays an important role. There has also been a well-documented positive association between large cell vasculitis and inflammatory AAA; in one case series of patients with Takayasu arteritis, there was an incidence of up to 45% [2].

While there are several theories, the exact pathogenesis of IAAA is unclear. Some argue that IAAA and atherosclerotic AAA are fundamentally similar. These proponents argue that IAAA is simply a severe inflammatory progression of the traditional atherosclerotic AAA. Others believe that IAAA is a result of a foreign antigen being introduced into the aortic wall, triggering an intense inflammatory cascade that ultimately damages aortic walls [3]. Additionally, other sources posit that IAAA is a direct result of infection.

While clinical presentation can vary, the most common presenting signs include weight loss, abdominal/back pain, and an elevated erythrocyte sedimentation rate (ESR). The most common imaging modality to evaluate IAAA is CT angiography. CTA accurately evaluates the anatomy of the aneurysm. In comparison to conventional angiography, CTA has the added advantage of evaluating surrounding structures. Common CT findings include dilation of aorta, aortic wall thickening, and surrounding soft tissue changes indicative of inflammatory processes. MRI, due to high-resolution visualization of the aorta, is also increasingly used. MRI will reveal the aortic mass as hypointense on T1-weighted images and hyperintense on T2-weighted images. MRI will also reveal dilation of aorta, thickened aortic wall, and soft tissue changes [4]. Ultrasound is frequently used as a screening modality for AAA and is recommended as an elective one-time screening for all men aged 65-75 with any smoking history. PET can also be used in conjunction with CT or MRI to evaluate disease activity.

Surgical repair of inflammatory AAA carries increased risk compared to repair in noninflammatory AAA, due to technical complications of extensive fibrosis and inflammation [5]. Nonsurgical treatment options consist of corticosteroids and immunosuppressive medication. However, there is speculation that steroidal use may increase the likelihood of aneurysm rupture. Furthermore, no studies have examined the long-term efficacy of steroidal use in management of IAAA. As a result, surgical intervention is still the treatment of choice.

## Conclusions

Inflammatory abdominal aortic aneurysms (IAAA) are a distinct yet dangerous subcategory of abdominal aortic aneurysms. Early diagnosis and treatment are critical to patient outcome.

## Patient consent

Written and informed consent was obtained for publication from the patient.
